# Functional recovery after spinal cord injury through neuroprotection by lipoic acid-loaded hollow mesoporous Prussian blue nanozymes

**DOI:** 10.1093/rb/rbag039

**Published:** 2026-03-09

**Authors:** Qiannan Zhao, Yuanlong Li, Jiaqi Zhang, Kai Gao, Lin Shi, Ensi Liu, Wenjuan Yang, Tingting Zhang, Xifan Mei, Zhaoliang Shen

**Affiliations:** Department of Orthopedics, Third Affiliated Hospital of Jinzhou Medical University, Jinzhou, Liaoning 121001, P. R. China; Liaoning Provincial Collaborative Innovation Center for Medical Testing and Drug Research, Jinzhou, Liaoning 121001, P. R. China; Key Laboratory of Medical Tissue Engineering of Liaoning Province, Jinzhou Medical University, Jinzhou, Liaoning 121001, China; Orthopedics, Sun Yat-Sen Memorial Hospital, Sun Yat-sen University, 107 Yanjiangxi Road, Guangzhou, Guangdong 510120, P. R. China; Department of Orthopedics, The First Hospital of China Medical University, No. 155 NanjingBei Street, Heping District, Shenyang, Liaoning Province 11000, P. R. China; Department of Orthopedics, Jining No. 1 People’s Hospital, Jining 272011, P. R. China; Department of Orthopedics, Third Affiliated Hospital of Jinzhou Medical University, Jinzhou, Liaoning 121001, P. R. China; Liaoning Provincial Collaborative Innovation Center for Medical Testing and Drug Research, Jinzhou, Liaoning 121001, P. R. China; Key Laboratory of Medical Tissue Engineering of Liaoning Province, Jinzhou Medical University, Jinzhou, Liaoning 121001, China; Department of Orthopedics, Third Affiliated Hospital of Jinzhou Medical University, Jinzhou, Liaoning 121001, P. R. China; Liaoning Provincial Collaborative Innovation Center for Medical Testing and Drug Research, Jinzhou, Liaoning 121001, P. R. China; Key Laboratory of Medical Tissue Engineering of Liaoning Province, Jinzhou Medical University, Jinzhou, Liaoning 121001, China; Department of Pathology, First Affiliated Hospital of Jinzhou Medical University, Jinzhou, Liaoning 121001, P. R. China; The Eighth Affiliated Hospital, Sun Yat-sen University, Shenzhen, Guangdong 518033, P. R. China; Department of Orthopedics, Third Affiliated Hospital of Jinzhou Medical University, Jinzhou, Liaoning 121001, P. R. China; Liaoning Provincial Collaborative Innovation Center for Medical Testing and Drug Research, Jinzhou, Liaoning 121001, P. R. China; Key Laboratory of Medical Tissue Engineering of Liaoning Province, Jinzhou Medical University, Jinzhou, Liaoning 121001, China; Liaoning Vocational College of Medicine, Shenyang, Liaoning 110100, China; Department of Orthopedics, Third Affiliated Hospital of Jinzhou Medical University, Jinzhou, Liaoning 121001, P. R. China

**Keywords:** spinal cord injury, Prussian blue nanozyme, lipoic acid, ROS, neuronal protection, Keap1/Nrf2

## Abstract

The key obstacle to functional recovery after spinal cord injury (SCI) is the imbalance of the oxidative stress microenvironment in the injured area. Traditional drug therapies have limitations in regulating this environment and eliminating the excessive accumulation of reactive oxygen species (ROS) is crucial. In this study, an environmentally friendly and economical recombinant nanoenzyme (LA-HMPB) was successfully constructed, which achieves delivery to the SCI injury site and enhances the therapeutic capacity of lipoic acid (LA). This nanoenzyme alleviates oxidative stress through the Keap1/Nrf2 pathway, thereby promoting functional recovery after SCI. The research found that HMPB not only serves as a carrier but also enhances the antioxidant stress capacity of LA. After administration, LA-HMPB can distribute to the SCI site and exert its effects. It has been confirmed that this formulation reduces oxidative stress levels by regulating the Keap1/Nrf2 pathway, thereby promoting functional recovery. This natural nano-drug delivery platform strategy opens up broad prospects for the clinical treatment of SCI and provides a useful reference for the research on antioxidant therapy for other neurological diseases.

## Graphical Abstract

**Graphical Abstract:**
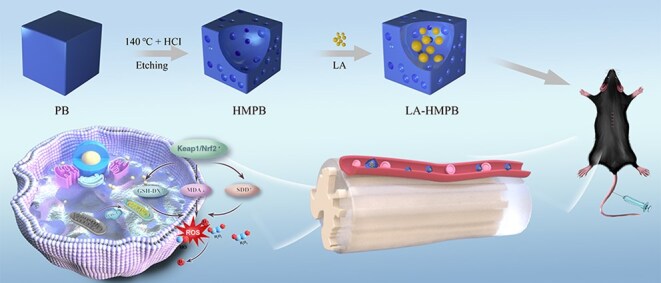

## Introduction

The causes of spinal cord injury (SCI) were divided into traumatic and non-traumatic [1]. After injury, the motor, sensory and autonomic nerve functions below the injury level had phased disorders [2]. The incidence of SCI is as high as 10.4–83 cases/million/year worldwide, which poses a serious burden to families of patients [3]. Although we have a certain understanding of the pathological basis and pathogenesis of SCI, there are still some limitations in nerve repair. SCI is a severe central nervous system disorder that leads to significant neurological impairments and is regarded as one of the most challenging prognostic diseases in clinical practice [4].

Furthermore, the prognosis of SCI is primarily contingent upon the quantity and status of neurons [5]. Neurons are the core of signal transduction in the spinal cord, and their damage leads to the interruption of nerve conduction and loss of function. Mitochondria are the key organelles for the energy supply of neurons, and their dysfunction can directly weaken the metabolic basis of neurons, thereby aggravating the decline of nerve conduction function [6, 7]. After injury, mitochondrial dysfunction triggers the activation of many oxidase enzymes, leading to the production of large amounts of reactive oxygen species (ROS) and reactive nitrogen species (RNS), which damage neurons [8, 9]. Hence, it is of paramount importance to safeguard the damaged neurons by resolving mitochondrial dysfunction and ameliorating the oxidative stress microenvironment. Due to the extremely low ability of nerve cells to regenerate after SCI, this presents a major therapeutic dilemma for clinicians [10]. Despite the widespread clinical use of anti-inflammatory and neuroprotective agents, these agents are often ineffective in the intervention of mitochondrial diseases, so there is an urgent need to develop intervention methods in this direction [11, 12].

The potential of hollow Prussian blue nanozymes (HMPB) is increasingly acknowledged in the evolving field of nanomedicine [13, 14]. HMPB has attracted much attention for free radical scavenging and drug delivery due to its inherent enzyme-like activity, adjustable high specific surface area and large pore volume [15, 16]. Despite their promising properties, the therapeutic applications of these materials have not yet been thoroughly investigated. Certain HMPB demonstrated promising anti-inflammatory and antioxidant properties, indicating their potential use in SCI treatment [17, 18]. Despite advancements, HMPB still faces significant challenges in catalytic efficiency, toxicity management and structural stability, hindering its practical application in biological systems [19, 20]. Enhancing the biocompatibility of HMPB and elucidating their interactions with biological pathways remain key areas of research.

**Figure 1:**
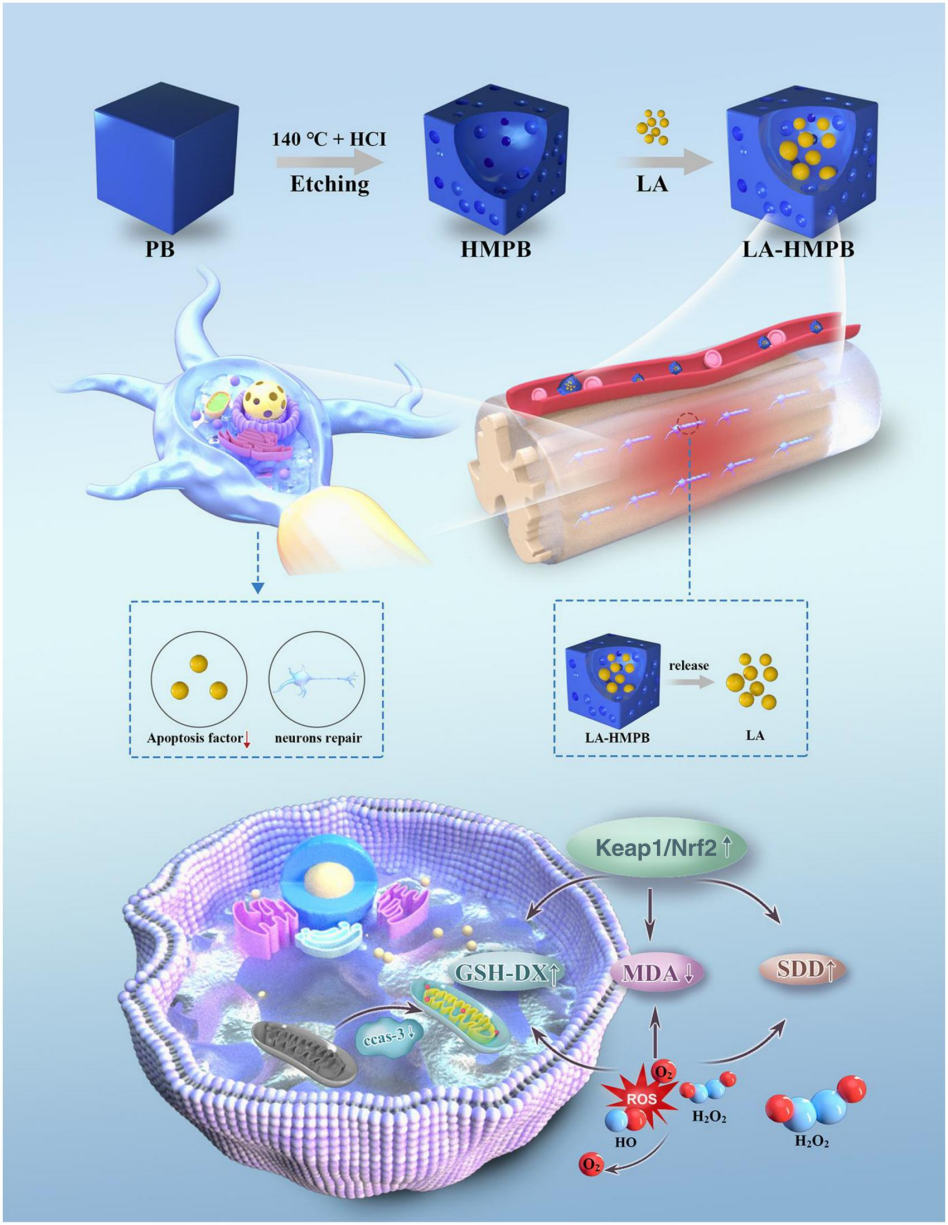
Schematic of the mechanism by which lipoic acid-loaded hollow mesoporous Prussian blue nanozyme accelerate recovery after spinal cord injury.

The hollow nature of HMPB is suitable for drug delivery and has been used as a drug carrier in many diseases. As a natural antioxidant, lipoic acid (LA) has been recognized by researchers for its antioxidant capacity [21, 22]. To improve LA bioavailability and address the drawbacks of standalone HMPB treatment, we fabricated lipoic acid-encapsulated hollow mesoporous Prussian blue nanozymes (LA-HMPB) based on the complementary advantages of HMPB as an efficient drug carrier and LA as a robust antioxidant (Figure 1). This study aims to clarify whether LA-HMPB modulates oxidative stress and suppresses neuronal apoptosis in both *in vitro* and *in vivo* SCI models. It also explores whether these effects are mediated via the Keap1/Nrf2 pathway, which serves as a key regulatory axis for neuronal apoptosis following SCI [23–25]. HMPB can promote the ability of LA to treat SCI and achieve the purpose of synergistic treatment. This study reveals a novel therapeutic approach for the treatment of SCI by *in vivo* targeted delivery.

## Materials and methods

### Synthesis of HMPB and LA-HMPB

The synthesis of HMPB was optimized based on previously reported methods [26]. The detailed procedures were as follows: Initially, 8.0 g of polyvinylpyrrolidone (PVP, Sigma-Aldrich, St. Louis, MO, USA) was dissolved in 50 mL of 1 M hydrochloric acid solution (HCl, Adamas-beta, Shanghai, China). Subsequently, 700 mg of potassium hexacyanoferrate(III) (K_3_[Fe(CN)_6_], Adamas-beta) was added to the solution. The mixture was magnetically stirred at room temperature for 1 h and then transferred to an electric heating constant-temperature drying oven. The reaction was conducted at 100°C for 20 h. After completion of the reaction, the product was harvested by centrifugation. Then, the synthesized samples were centrifuged (10 000 rpm, 15 min) and washed with ethanol and distilled water for three times, redispersed in deionized water to form a homogeneous colloidal solution and stored at 4°C in the dark for subsequent use. For the preparation of LA-HMPB, the pre-synthesized HMPB was dissolved in chloroform to prepare a 10-mg/mL solution, which was then thoroughly mixed with LA chloroform solution at a volume ratio of 1:10. The chloroform solvent was removed via low-pressure rotary evaporation (40°C, 150 rpm), resulting in the formation of a uniform film on the container wall. Centrifugation (12 000 rpm, 8 min) was performed to collect the precipitates. After washing with PBS several times, the nanoparticles were obtained and dried, denoted as LA-HMPB, for further use.

### Material characterization

The morphology of LA-HMPB was detected by high-resolution Scanning Electron Microscope (SEM) (Hitachi, S4800, Tokyo). After ultrasonic dispersion, the samples were dropped onto carbon-supported copper mesh and dried naturally. The microscopic morphology of LA-HMPB was observed under an accelerating voltage of 15 kV. LA-HMPB was identified and characterized by Fourier Transform Infrared Spectroscopy (FTIR) spectroscopy. The LA-HMPB freeze-dried samples were ground to 2 μm, mixed with Potassium Bromide (KBr) and tabbed to analyze the characteristic functional group vibrational modes. The absorption spectra of each group were obtained separately, and their absorption spectra matched the corresponding vibration modes of each group. The same phrase has been repeated twice, and ‘diffraction instrument’ and ‘X-ray diffractometer’ refer to the same device. The duplicate section should be deleted and the text consolidated into a single sentence. The samples were dried, ground and then tableted in a range of 10–70° (2 θ). The hydrated particle size and zeta potential of HMPB and LA-HMPB were assessed by Dynamic Light Scattering (DLS) (Malvern Zetasizer Nano ZS, Germany). The kinetics of LA release from LA-HMPB at different pH conditions was determined by High-Performance Liquid Chromatography (HPLC). LA-HMPB (5 mg) was dispersed in 10 mL of PBS buffer (pH 5.0 or 7.4) and incubated at 37°C with constant shaking (100 rpm). The supernatant was sampled at specific time points, the cumulative sample release was quantified and the release curve was plotted. The subsequent supplementary experiments are sourced from Science Compass Company (China).

### Cell preparation

PC12 cells are a neuroendocrine cell line that originate from the adrenal medulla of rats and exhibit characteristics of both neurons and glial cells. Cell experiments were performed with PC12 cells cultured at 37°C and 5% CO_2_, utilizing high-glucose Dulbecco’s modified Eagle medium (DMEM) supplemented with 10% fetal bovine serum and 0.04% penicillin-streptomycin. PC12 cells were divided into H_2_O_2_ group, H_2_O_2_+LA group, H_2_O_2_ + HMPB group and H_2_O_2_+LA-HMPB group. Each group was exposed to the H_2_O_2_ (80 μg/mL), H_2_O_2_+LA (5 μg/mL) group, H_2_O_2_ + HMPB (5 μg/mL) group and H_2_O_2_+LA-HMPB (5 μg/mL) group in high-sugar medium of DMEM for 4 h, respectively. All the components of the cultured cells mentioned above are from Gibco.

### Cell proliferation and CCK8

In a sterile environment, the PC12 cell suspension was distributed into a 96-well culture plate, ensuring each well received 100 μL with a minimum cell density of 5 × 10^4^ cells. Then, the 96-well cell culture plate was placed into a cell incubator for 24 h, with all cell culture conditions maintained under standard settings (culture temperature of 37°C, CO_2_ concentration of 5%). Varying concentrations (1.25, 2.5, 5, 10 and 20 μg/mL) of HMPB and LA-HMPB were introduced to the culture wells. For LA, two sets of different concentrations were used for incubation in the cell incubator for 24 h: one set was (0, 1, 2, 4, 8 and 12 μg/mL), and the supplementary set of determined concentrations was (0, 4, 5, 6, 7 and 8 μg/mL). To eliminate the possibility of drug residue, the old cell culture medium was replaced with fresh medium. The medium was discarded, and medium containing 10% CCK-8 reagent was added to each well (Solarbio, CA1210, China). After incubation for 4 h, the absorbance was measured.

Cell viability was assessed using the formula: Cell viability (% of control) = (OD test—OD blank)/(OD control—OD blank).

Among them, the absorbance of the experimental group OD test was at 450 nm, the absorbance of the blank group was OD blank and the absorbance of the control group was OD control.

### Animals preparation

Eighty 8-week-old C57BL/6 mice, with equal numbers of males and females, weighing around 20 ± 0.6 g, were utilized. They were purchased from Liaoning Changsheng Biotechnology Co. Ltd, China. The laboratory mouse was raised under specific pathogen-free (SPF) conditions at the Animal Experimental Center of Jinzhou Medical University. Each cage can hold up to six mice at most. Two weeks were used to acclimate the animals before raising them to ensure that they adapted to the new environment. All animal experiments adhered to the guidelines approved by the Animal Care Committee and Jinzhou Medical University [Approved Ethic Number: SYXK 2024-0012].

### Preparation of animal models

A mouse model for SCI was created using an intraperitoneal injection of 1% sodium pentobarbital at a dosage of 50 mg/kg. After anesthesia, the skin was prepared for disinfection. Then a precise incision was made in the sterilized area to expose the T9-T10 level for laminectomy. A 10 g, 2 mm diameter calibrated impactor caused the contusion by falling 2 cm onto the exposed spinal cord. In the sham operation group, only laminectomy was performed without inducing spinal cord contusion injury. The mice were then placed in a 37°C incubator to aid in the recovery of anesthesia. To ensure normal voiding function, bladder massage was performed twice daily while their overall health was closely monitored. In a sterile environment, strict hygiene standards were adhered to with thorough disinfection procedures for all instruments. The mice were divided into four groups: SCI+Saline group, SCI+LA group, SCI+HMPB group and SCI+LA-HMPB group. Three hours post-SCI model establishment, tail vein injections were administered as follows: Saline (5 mg/kg), LA (5 mg/kg), HMPB (5 mg/kg) and LA-HMPB (5 mg/kg); 0.1 mL of the liquid formulation was injected at the same indicated time each day.

### Test of hemolysis

Hemolysis test was used to evaluate the safety of LA and LA-HMPB *in vivo*. Fresh mouse blood was washed with PBS until the supernatant became clear and no red blood cells were visible. Next, diluted the red blood cells to 2% (V: V) with PBS. Different concentrations of LA and LA-HMPB (1.25, 2.5, 5, 10 and 20 μg/mL) were mixed with 2% red blood cells. The mixture was allowed to rest for 3 h at room temperature and centrifuged at 10 000 rpm for 3 min. Finally, the supernatant (100 μL) was removed to a 96-well plate and incubated at 570 nm using a microplate reader (Versa Max, Molecular Devices, Sunnyvale, CA, USA). Positive and negative controls corresponded to water and PBS, respectively. Each sample was analyzed three times in parallel. The hemolysis percentage was calculated by the following formula:


Hemolysis percentage(%)=[(A sample−A negative control)/(A positive control−A negative control)]×100%

A hemolysis rate of >5% is regarded as hemolysis.

### Testing of liver and kidney function

After 28 days of treatment, blood samples were collected from each mouse and placed in 1.5 mL centrifuge tubes. The blood was allowed to stand for 45 − 65 min before being centrifuged at 4°C and 5000 rpm for 10 − 15 min to separate the serum. The serum was subjected to an additional 3-min centrifugation. The serum was placed in a centrifuge tube, and a biochemical analyzer was employed to statistically assess aminotransferase (AST), alanine aminotransferase (ALT), creatinine (CRE) and blood urea nitrogen (BUN).

### Detection of reactive oxygen species

The cultured PC12 cells were treated with 50 μM H_2_O_2_ for 4 h. After the treatment, they were incubated with the corresponding nanomaterials and drugs for 24 h and then grouped. Next, used 1 mL of 2',7'-Dichlorodihydrofluorescein diacetate (DCFH-DA) (Solarbio, CA1410, 1:1000). Replace the original culture medium and continue incubating for 30 min. After the incubation was completed, the cells were washed three times with the original culture medium, each washing for 5 min. Finally, the fluorescence intensities of cells in each group were recorded under the microscope.

### Measurement of mitochondrial membrane potential

PC12 cells were evenly spread into six-well plates and the Mitochondrial Membrane Potential Assay kit (JC-1) (Solarbio, M8650, China) was used according to the company’s product instructions. At the end of cell incubation, the fluorescence intensity was observed and recorded under a fluorescence microscope.

### Oxidative stress test

At least 5 × 10^4^ cells were collected for analysis. The cellular levels of malondialdehyde (MDA), superoxide dismutase (SOD) and reduced glutathione (GSH) were measured using specific assay kits. The total antioxidant capacity assay kit (Beyotime Biotechnology) was used to measure the trolox equivalent antioxidant capacity (TEAC), while the MDA, SOD and GSH content assay kits (Solarbio, BC0020, BC0175, BC1175, China) were applied for the determination of MDA, SOD and GSH, respectively.

Tissue samples were collected from 0.5 cm above and below the SCI site in mice. The tissue levels of MDA, SOD and GSH were measured following the instructions of the corresponding assay kits.

### Flow cytometry

To measure the apoptosis rate of PC12 cells, the Transwell co-culture system was removed after each group of material was treated, the upper PC12 cells were resuspended and 100 000 PC12 cells were mixed in 100 μL of binding buffer. The final assay was performed to observe apoptosis (Beyotime, C2015M, China).

### Cell immunofluorescence staining assay

PC12 cells in each group were pre-treated with H_2_O_2_ for 4 h in confocal dishes (biosharp, BS-20-GJM, China), then treated with respective reagents for 12 h under controlled conditions. Cells were fixed with 4% paraformaldehyde, permeabilized with 0.3% Triton X-100, blocked with 5% goat serum for 2 h and incubated overnight with primary antibodies: β-tubulin (CST, 1:500, 2146, USA), Cleaved caspase-3 (Affinity, 1:500, AF7022, China), Bax (Affinity, 1:500, AF6931, China), Bcl-2 (Affinity, 1:500, AF0769, China), HO-1 (Affinity, 1:500, DF6391, China), NeuN (Affinity, 1:500, DF6145, China). After three washes, samples were incubated for 2 h with Alexa Fluor 488 goat anti-rabbit IgG or Alexa Fluor 594 goat anti-mouse IgG (Thermo, 1:500, A-11012, USA), washed with PBS, stained with DAPI for 15 min, imaged via Leica TCS SP5II CLSM and fluorescence intensity quantified using ImageJ.

### Total RNA extraction and quantitative reverse transcription PCR

Total RNA was extracted from optimally cultured PC12 cells and C57BL/6 mouse spinal cords using Trizol/phenol/chloroform buffer. RNA purity (via OD260/OD280 ratio) and concentration (NanoDrop 2000) were verified for eligibility. Reverse transcription with PrimeScript RT Master Mix (Takara) generated cDNA for RT-qPCR. All steps followed the protocol; primers are in Supplementary Table S1.

### Tissue sections

Three days later, SCI mice were fully anesthetized via intraperitoneal injection of 1% pentobarbital. After modeling, cardiac perfusion was performed with 4% PFA. A 1-cm spinal cord segment at the injury site was immersed in 4% PFA for 48 h, then sequentially dehydrated in 10%, 20% and 30% sucrose solutions, and sectioned into 10 μm slices using a cryostat. For paraffin sections, mice were anesthetized and perfused as above, followed by spinal cord collection. Tissues were dehydrated in gradient ethanol (80, 90, 95 and 100%) and cleared with xylene.

### Tissue immunofluorescence

Frozen sections were air-dried for 1 h at room temperature, washed with PBS and incubated with 0.3% Triton X-100 for 15 min. After removing PBS, sections were blocked with 5% normal goat serum for 2 h (room temperature), then incubated with primary antibodies (Cleaved-caspase 3, NeuN; Solarbio, 1:500) at 4°C overnight. They were then incubated with secondary antibodies for 2 h, stained with DAPI and observed under a Leica TCS SP5 II fluorescence microscope.

### Western blot

Western blot was used to detect apoptosis-related proteins (Cleaved-caspase 3, Bcl-2, Bax, Keap1, Nrf2, β-tubulin). Spinal cords from each group were dissected on ice; 1 cm segments centered at the injury site were taken, and proteins extracted into 1.5 mL EP tubes. After concentration determination, proteins were separated by 10% SDS-PAGE, transferred to PVDF membranes, blocked with 5% skim milk for 2 h, then incubated with specific antibodies for 2 h. Primary antibodies were incubated at 4°C overnight, and secondary antibodies at room temperature for 2 h. Bands were visualized via BIO-RAD imaging system and analyzed for intensity using ImageJ.

### Imaging of drug distribution in organs

The drug was combined with fluorescein isothiocyanate (FITC) (Solarbio, F8070, China) using a magnetic mixer for 24 h before being administered to SCI mice. The distribution and metabolic pathways of the drugs *in vivo* were assessed using IVIS (PerkinElmer) imaging on the heart, liver, spleen, lung, kidney, brain and spinal cord at 0, 3, 6, 12 and 24 h post-injection.

### Behavioral assessment

Behavioral tests were administered between 8 and 10 am. The mice were brought to the laboratory 1 h before each test for acclimation.

### Footprint analysis

In order to detect the mobility of the mice’s hind limbs, footprint analysis was conducted for each group. Following a 28-day treatment period for each group of mice, their front limbs were marked with black dye and their back limbs with red dye.

### Basso mouse scale (BMS)

BMS was used to evaluate functional recovery of injured mice on Days 1, 3, 7, 14, 21 and 28 (BMS: 0–9 points). Mice from each group were placed in an open field, observed by three blinded researchers for 4 min (assessing ankle/paw/dorsal foot contact, trunk stability, tail position) and the experiment was repeated three times.

### Weight monitoring

After modeling, mice were divided into sham operation, Saline, LA, HMPB and LA-HMPB groups, with each group injected with the corresponding drug. Mouse body weights were measured and recorded at 0, 7, 14 and 28 days.

### Slope test

Mice were placed on a wooden slope with heads facing the higher end. The slope angle was gradually increased, and the maximum angle at which mice could hold for 5 s was recorded. A larger angle indicated stronger hindlimb weight-bearing ability.

### Rotarod test

Mouse motor ability was evaluated via the rotarod test. After 28 days of treatment, mice were placed on a Ugo Basile rotarod (model 47650, Gemonio, Italy) with a 2-cm diameter rod rotating at 5 rpm (1 rpm acceleration) parallel to sea level. The duration until falling was recorded.

### Hematoxylin−eosin (HE) staining

5-µm-thick paraffin sections were prepared from major organs/tissues (heart, liver, spleen, lung, kidney, spinal cord) of mice in each group. HE staining was performed per instructions, followed by microscopic observation and imaging for further analysis.

### Nissl staining

Spinal cord tissues from each mouse group were prepared and sectioned into 5 μm paraffin slices. The Nissl staining was conducted following the methylene blue method as per Solarbio’s instructions, and images were documented using a microscope.

### Statistical analysis

Experimental data are presented as mean ± SD. Inter-group differences were analyzed via one-way ANOVA and Tukey’s multiple comparison test using GraphPad Prism software. *P* < 0.05 was considered statistically significant.

## Results

### Characterization of LA-HMPB and determination of loaded LA

**Figure 2:**
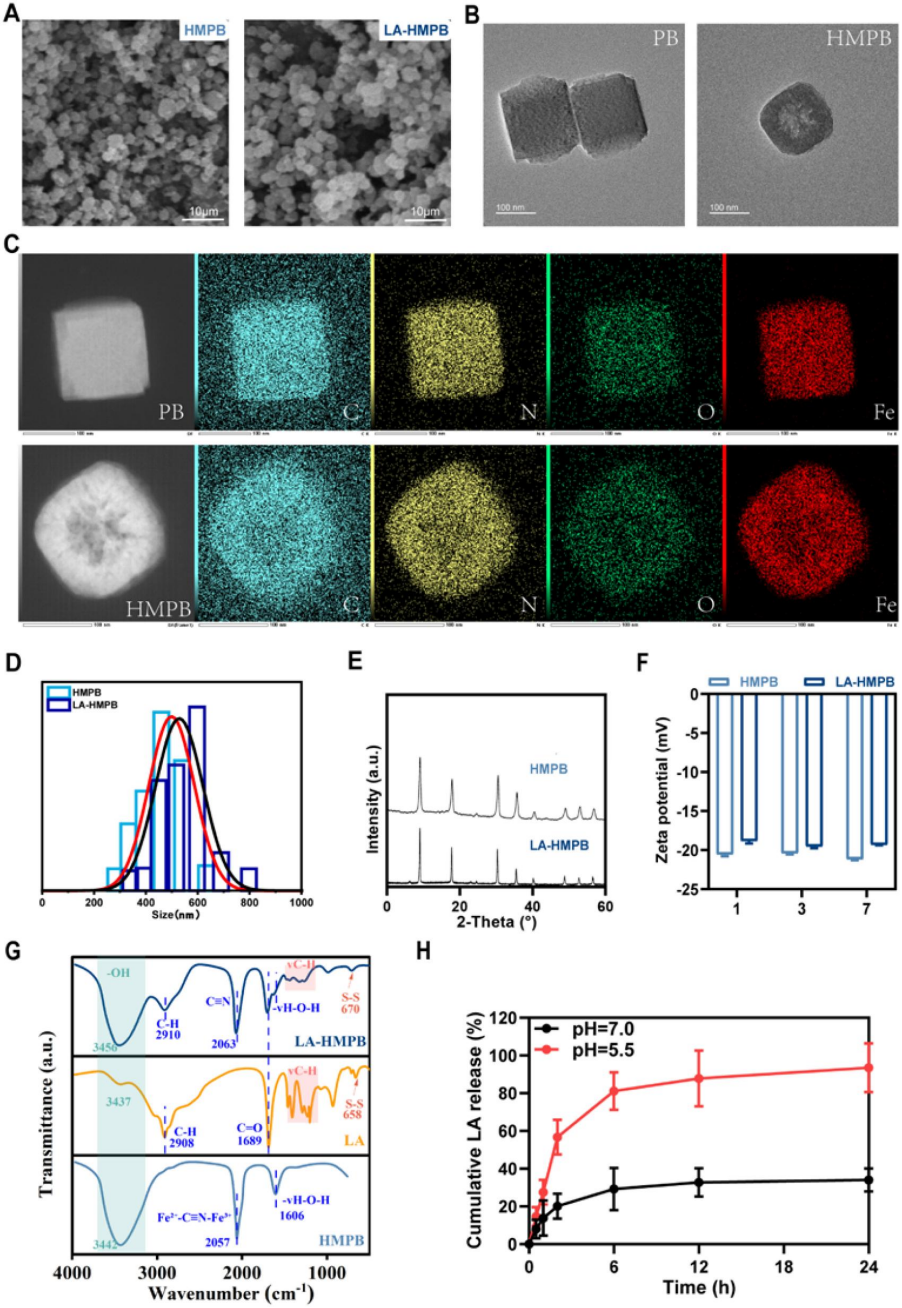
Characterization of LA-HMPB and determination of loaded LA. (**A**) SEM images of HMPB and LA-HMPB at 10 μm magnification. (**B**) TEM images of PB and HMPB at 100 nm. (**C**) Elemental mapping of PB and HMPB. (**D**) Particle size analysis test of LA-HMPB and HMPB. (**E**) X-ray diffraction test. (**F**) Zeta potential. (**G**) LA, HMPB and LA-HMPB infrared absorption spectroscopic testing. (**H**) *In vitro* drug release of LA under different pH conditions.

Preliminary observations via SEM demonstrate the characteristics of HMPB and LA-HMPB, with uniformly distributed nanoparticles clearly visible. Moreover, after LA loading, the spatial structure of HMPB remains unchanged, and LA-HMPB still maintains its hollow cubic structure (Figure 2A). To further verify the successful synthesis of HMPB, we conducted TEM analysis on PB and HMPB particles. The results showed that both types of particles exhibit a cubic structure in terms of morphology; in contrast, hollow features can be clearly observed in HMPB particles, and this phenomenon further confirms our previous hypothesis that HMPB has a hollow structure (Figure 2B). The elemental mapping results of PB and HMPB indicate that both contain four elements: Fe, C, N and O. In terms of morphological characteristics, both exhibit a cubic structure. The consistency in elemental composition and morphology confirms that PB and HMPB are nanoparticles with the same matrix. Meanwhile, by analyzing the distribution characteristics of fluorescent signals, the hollow structure of HMPB is also indirectly verified (Figure 2C). SEM images of HMPB and LA-HMPB nanoparticles showed a cubic and similarly uniform-sized structure, with an average diameter of 16.7 nm for HMPB and 15.97 nm for LA-HMPB (Figure 2D). XRD spectra show that LA-HMPB retains the cubic structure of HMPB after carrying LA (Figure 2E). Additional analysis was conducted to assess the zeta potential of HMPB and LA-HMPB. The decreased zeta potential of LA-HMPB compared to HMPB indicates successful LA incorporation into HMPB (Figure 2F). FTIR results showed that LA-HMPB retained the characteristic C≡N peak of HMPB at 2063 cm^−1^ and the S-S peak of LA at 670 cm^−1^, while the C = O peak of LA at 1689 cm^−1^ was absent, confirming the successful conjugation of LA and HMPB (Figure 2G). *In vitro* drug release experiments were conducted at pH 5.5 and 7.0 to assess release profiles in acidic and neutral environments, reflecting the acidic microenvironment and inflammatory cells in SCI lesions. At pH 7.0, LA-HMPB released 29.2% of LA within the first 6 h. At pH 5.5, 81.1% of LA is released from LA-HMPB. Although the release was high, it only reached 93.5% in the next 18 h (Figure 2H). This indicates that the drug release begins quickly and then decelerates as the drug partially adheres to the HMPB surface instead of being completely encapsulated within the core. The above results can prove that LA is successfully loaded on HMPB, and LA-HMPB has unique advantages in drug release, which provides strong support for follow-up experiments.

### Physicochemical properties and bio-toxicity testing of lipoic acid-loaded Prussian blue nanozyme particles

**Figure 3:**
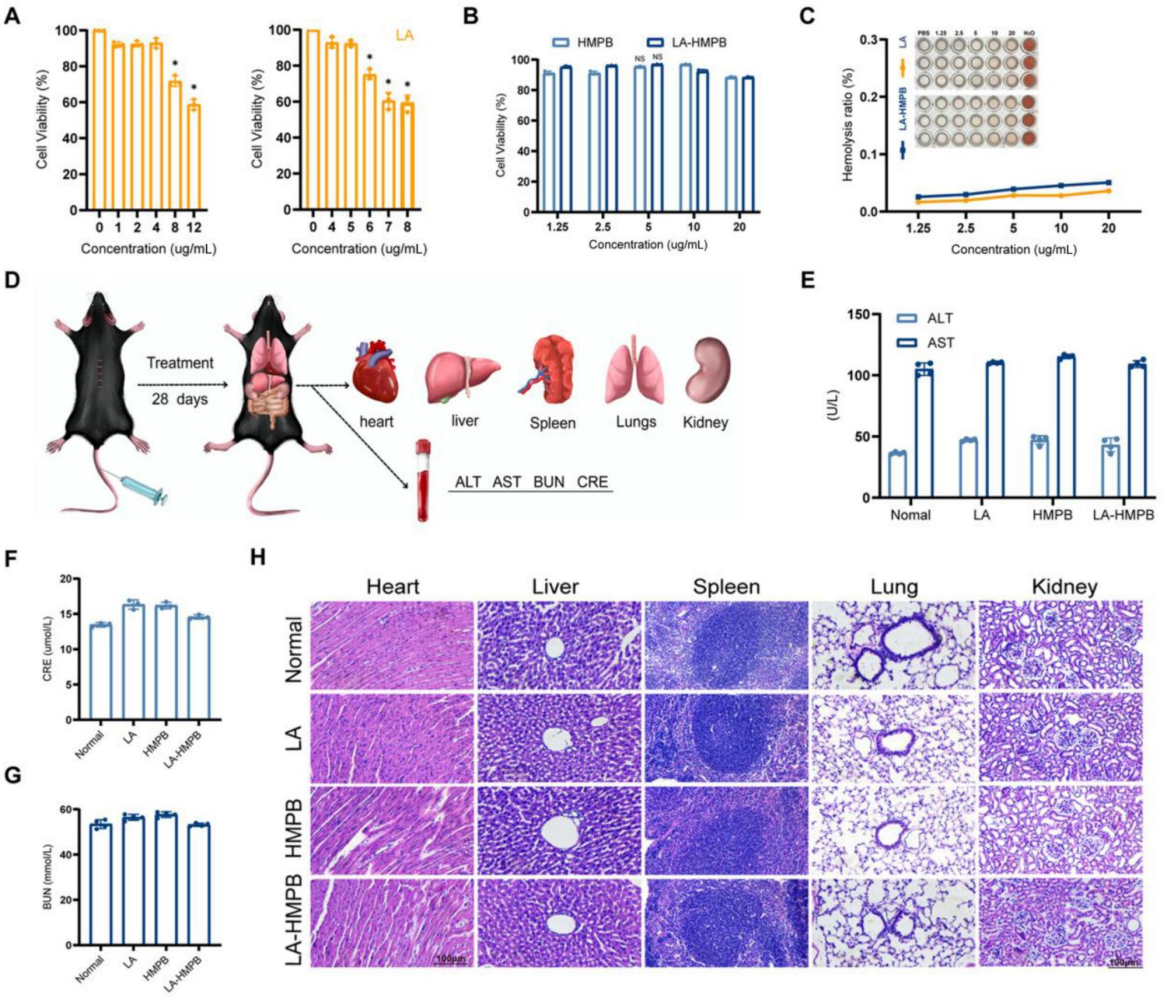
Drug safety was assessed *in vitro* and *in vivo*. (**A** and **B**) The cell viability of PC12 cells treated with LA (**A**), HMPB and LA-HMPB (**B**) for 24 h. (**C**) After tail vein injection of LA and LA-HMPB, hemolysis experiments were performed. (**D**) Schematic of major organs (heart, liver, spleen, lung and kidney) and biochemical examinations collected after 28 days of treatment. (**E–G**) Evaluation of liver function markers (AST, ALT) and renal function markers (Cr, BUN) in normal mice and mice treated for 28 days post-SCI. (**H**) HE staining of the heart, liver, spleen, lung and kidney in normal mice and mice treated 28 days after SCI, scale bar = 100 μm. All data are presented as mean values ± SD (*n* = 3): *P < 0.05, **P < 0.01.

Ensuring the safety of drugs is crucial for maximizing their therapeutic potential. We initiated a comprehensive assessment of the biocompatibility of LA, HMPB and LA-HMPB. Our thorough experiments assessed the effects on cell viability, hemolysis and hepato-renal toxicity, demonstrating remarkable biosafety [27, 28]. Rat chromaffin cell tumor cells (PC12 cells) are commonly utilized as a neuronal model for SCI treatment due to their neuronal characteristics. We initially assessed the viability of PC12 cells to investigate the cytotoxic effects of nanoparticles, including LA, HMPB and LA-HMPB. As shown in Figure 3A, based on the results of the LA cell viability experiment, where no significant effects were observed at low concentrations (1, 2 and 4 μg/mL), 5 μg/mL is identified as the critical concentration that is both effective and low-toxic. It can form a clear dose-effect window between the ‘no-effect range’ and the ‘high-toxicity range’, thus being selected as the experimental concentration. According to the data on PC12 cell viability determined by LA, we loaded 5 μg/mL of LA onto the HMPB. Both types of PB-based nanoparticles exhibited low toxicity at concentrations below 20 μg/mL (Figure 3B). In summary, the cytotoxicity tests suggest that LA-HMPB exhibits good biocompatibility in the acute phase. Oxidative stress-induced neuronal death is a primary contributor to secondary injury exacerbating SCI [29]. The study utilized H_2_O_2_ to mimic oxidative damage from SCI and evaluate the neuroprotective effects of LA-HMPB on neuronal survival and function. Additionally, co-incubation experiments with red blood cells were conducted to assess the potential toxicity of LA-HMPB and LA. Within 12 h, the hemolysis rate for both LA and LA-HMPB remains under 5%, suggesting a negligible hemolytic effect (Figure 3C). As shown in Figure 3D, blood samples were collected and relevant visceral organs were harvested from mice in different treatment groups after 28 days of intervention for subsequent experiments. We evaluated the liver and kidney toxicity of the drug in mice over 28 days by analyzing key markers such as AST, ALT, CRE and BUN. The findings were reassuring, showing no signs of liver or kidney toxicity (Figure 3E–G). Moreover, to further fortify the safety evaluation, we conducted HE staining on crucial organs such as the heart, liver, spleen, lungs and kidneys (Figure 3H). To our gratification, no notable pathological alterations were observed in any group, clearly validating the special biocompatibility of LA-HMPB synthesized in our study. These findings augment confidence in the safe use of LA-HMPB and further enhance its therapeutic potential.

### Protect mitochondria against oxidative stress

**Figure 4:**
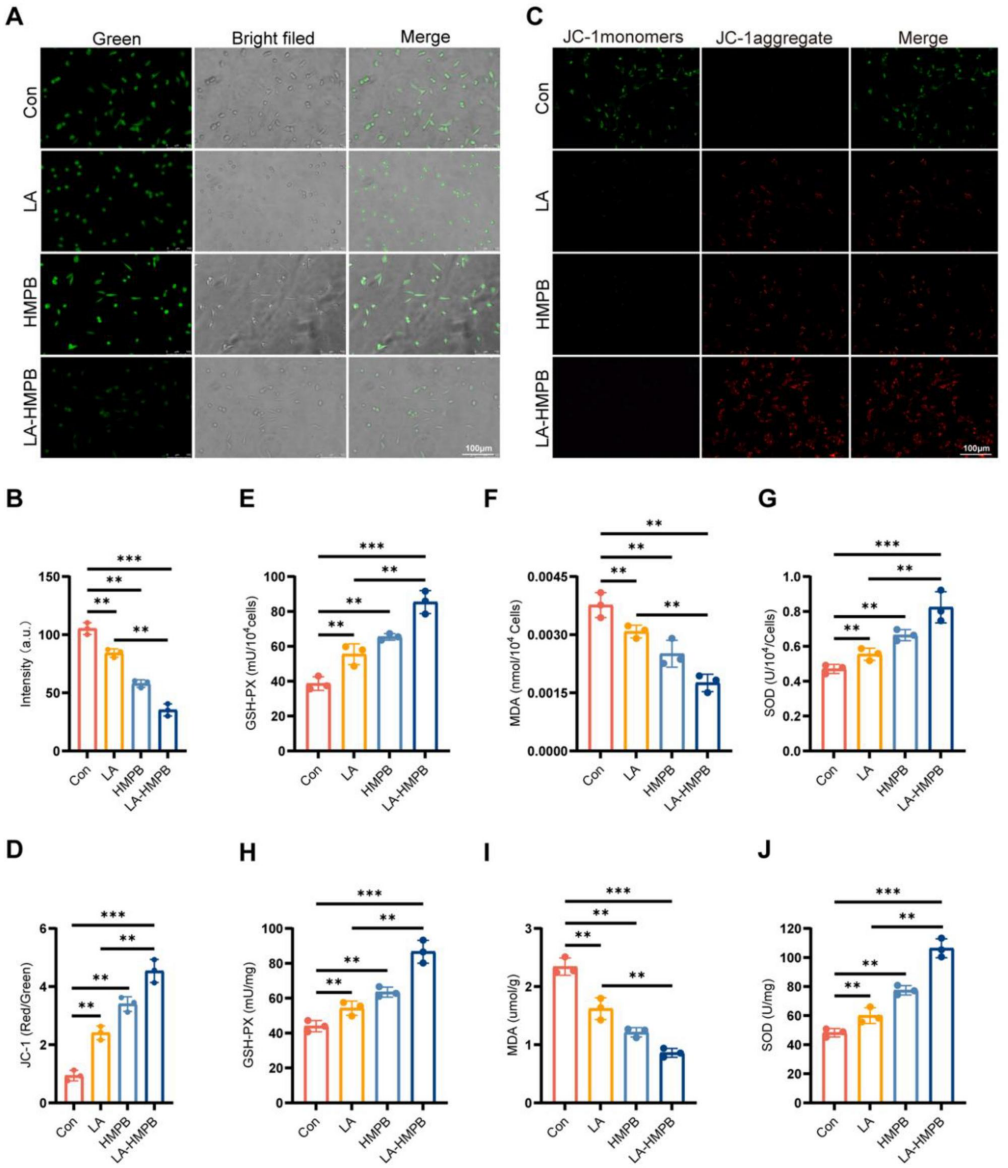
LA-HMPB Balances the oxidative stress microenvironment by protecting mitochondria. (**A** and **B**) Assessment of ROS levels in PC12 cells of the Con group, LA group, HMPB group and LA-HMPB group using the DCFH-DA ROS fluorescence probe, with quantification of green fluorescence intensity using statistical methods, scale bar = 100 μm. (**C** and **D**) Analysis of mitochondrial membrane potential in PC12 cells of each group through JC-1 staining, with quantification of the ratio of red light fluorescence intensity to green light fluorescence intensity using statistical methods. (**E–J**) The MDA level, SOD activity and GSH activity of PC12 cells and spinal cord tissue in each group were determined. All data are presented as mean ± SD (*n* = 3). *P < 0.05, **P < 0.01, ***P < 0.001.

After SCI, the subsequent imbalance of oxidative stress microenvironment affects mitochondrial function and neuronal viability. PC12 has all the characteristics of cells of the nervous system and is often used to study neurophysiological effects *in vitro*. After H_2_O_2_ treatment, the oxidative microenvironment was formed. To this end, we examined several oxidative stress key enzymes, ROS expression and mitochondrial membrane potential in PC12 cells [29, 30]. The expression of ROS probe (DCFH-DA) in the treatment group was lower than that in the Con group. Among the three treatment groups, although LA and HMPB showed a reduction in probe fluorescence, it was the LA-HMPB group that showed the most significant reduction (Figure 4A and B). LA-HMPB demonstrated a similar advantage in mitochondrial membrane potential (JC-1). Compared with the LA and HMPB groups, the expression of LA-HMPB was the weakest under green light and the most obvious under red light (Figure 4C and D). Due to the disruption of internal environment under oxidative stress, we further validated the measurement results of SOD, GSH-Px and MDA both *in vitro* and *in vivo*. Compared with the Con group, SOD and GSH-Px in each treatment group showed a tendency to increase, while MDA decreased to a certain extent (Figure 4E–G). In the *in vivo* experiment, the comparison of each treatment group with the Saline group was consistent with the *in vivo* experiment. Notably, in both *in vivo* and *in vitro* experiments, all three treatment groups were observed to exhibit a certain degree of antioxidant capacity. Among them, the LA group and HMPB group showed improvements in indicators detected by the oxidative stress assay kit; however, compared with the LA-HMPB combination treatment group, there is still room for improvement in their efficacy (Figure 4H–J).

### *In vitro* anti-neuronal apoptosis

**Figure 5:**
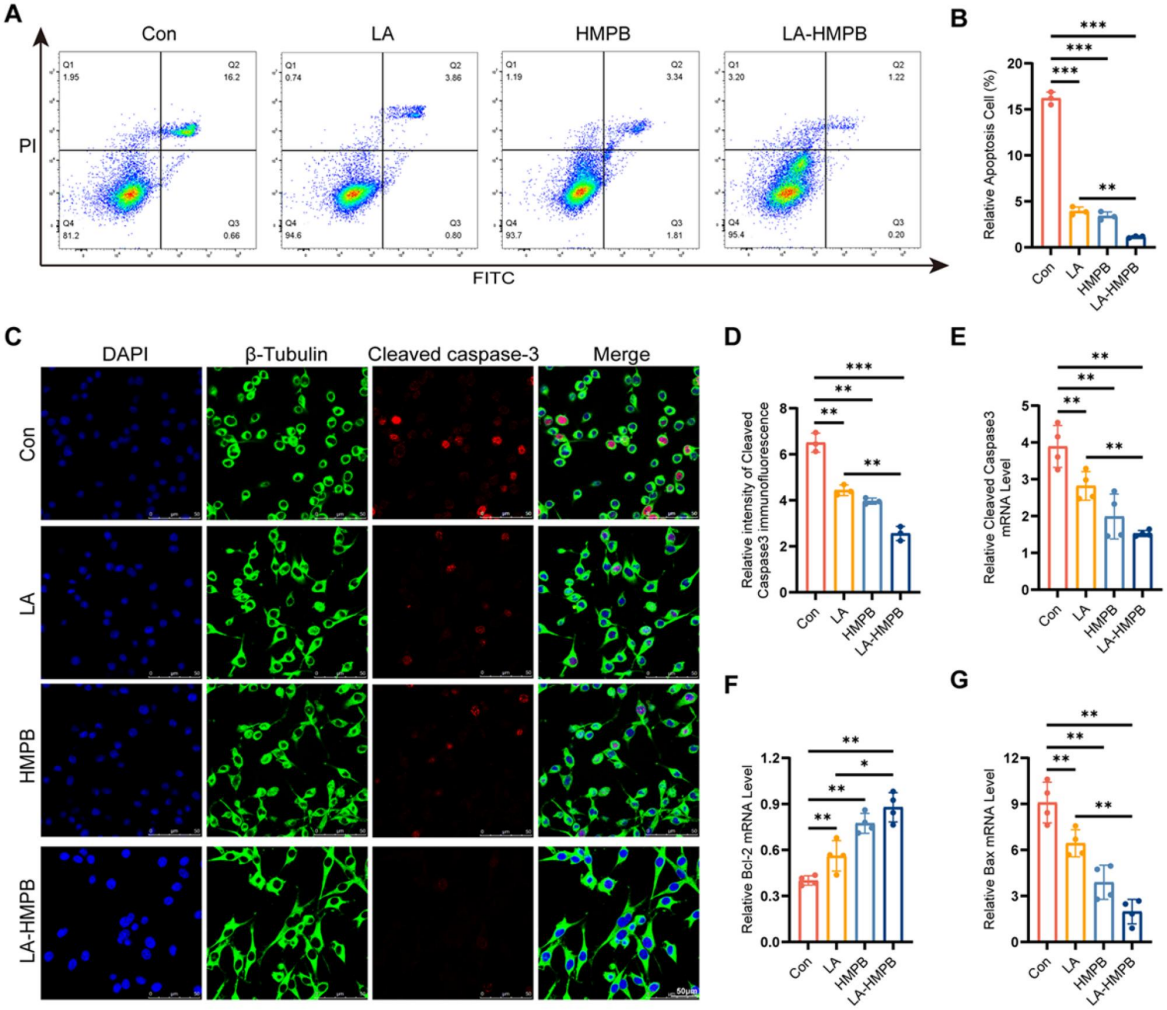
Evaluation of anti-apoptotic effects *in vitro*. (**A** and **B**) Apoptosis rates of the Con group, LA group, HMPB group and LA-HMPB group were measured using Annexin V-FITC apoptosis detection kit and flow cytometry, followed by quantitative analysis. (**C** and **D**) Immunofluorescence was employed to evaluate the expression of Cleaved-caspase 3 in PC12 cells across various groups, scale bar = 50 μm. Both the fluorescence intensity and the proportion of cells positive for Cleaved-caspase 3 were quantitatively assessed. (**E–G**) Relative Cleaved-caspase 3, Bax and Bcl-2 mRNA levels in PC12 cells of each group. All data are presented as mean values ± SD (*n* ≥ 3): *P < 0.05, **P < 0.01, ***P < 0.001.

We found that LA-HMPB possessed excellent antioxidant stress resistance [31]. Oxidative stress generated a large amount of ROS, which promoted cell apoptosis and led to neuronal damage. To verify whether it also had a certain inhibitory effect on cell apoptosis, we used PC12 cells *in vitro* to test our hypothesis. Known studies had shown that Bax was a protein that promoted apoptosis and was located in the cytoplasm [32, 33]. Bcl-2 was a protein that could restrain cell apoptosis and protect neuronal mitochondria. However, when the Bax protein content exceeded Bcl-2, it inhibited the growth of normal cells. It was worth noting that Bax and Bcl-2 both played irreplaceable roles in cell growth and development [34]. As mitochondrial function declined, cytochrome C was released into the cytoplasm, triggering a cascade of caspases that resulted in apoptosis [35]. Using the Annexin V-FITC Apoptosis Detection Kit, we observed that H_2_O_2_-treated PC12 cells exhibited a significant apoptosis rate of 16.2%. In contrast, the apoptosis rates for PC12 cells treated with LA, HMPB and LA-HMPB were reduced to 3.86, 3.12 and 1.22%, respectively, demonstrating the effective anti-apoptotic properties of LA-HMPB (Figure 5A and B). Cleaved-caspase 3 was a key performer in apoptosis, and was activated and played a central role in the process [36]. Next, we confirmed how LA, HMPB and LA-HMPB inhibited neuronal cell apoptosis. First, we adopted cell fluorescence technology to simulate and assess neuronal apoptosis. Our experimental results showed that in the neuronal cells treated with H_2_O_2_, the fluorescence intensity of Cleaved-caspase 3 increased, demonstrating that it was activated and the positive rate increased significantly (Figure 5C and D). In addition, we chose the RT-PCR technology in this experiment. We chose specific primers (Bax, Bcl-2 and Cleaved-caspase 3). According to the analysis of the results, the PCR results also confirmed that LA-HMPB had the effect of anti-neuronal apoptosis. Compared with the Con group, the mRNA expressions of Cleaved-caspase 3 and Bax in the treatment group showed a decreasing trend, while the mRNA levels of Bcl-2 showed an increasing trend. As in the previous study, the effect was most pronounced in the LA-HMPB group, whereas LA and HMPB groups were much less effective (Figure 5E–G).

### *In vivo* experiments to reduce neuronal apoptosis

**Figure 6:**
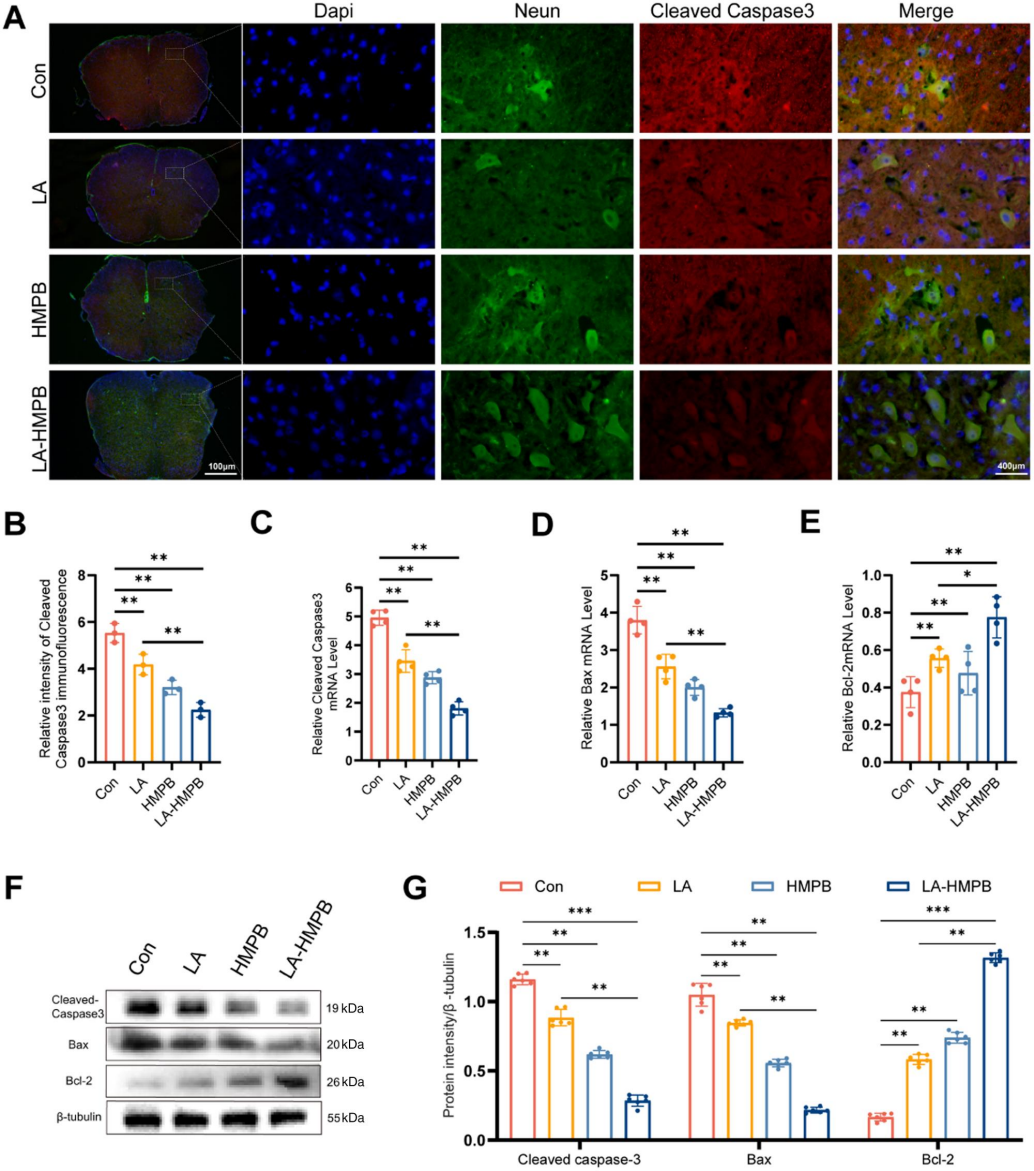
Anti-apoptotic effects of drugs in mice. (**A** and **B**) Staining of neuron and Cleaved-caspase3 fluorescence to determine the expression of Cleaved-caspase 3 in the spinal cord, with quantitative analysis of fluorescence intensity, scale bar = 400 μm. (**C–E**) Relative Cleaved-caspase 3, Bax, Bcl-2 mRNA levels in mice. (**F** and **G**) Western blot was used to analyze the expression of Cleaved-Caspase 3, Bax and Bcl-2 proteins in the spinal cord of each group, along with quantitative analysis of their grayscale values. All data are presented as mean values ± SD (*n* ≥ 3): *P < 0.05, **P < 0.01, ***P < 0.001.

In *in vitro* experiments, LA-HMPB has been observed to balance the oxidative stress microenvironment and inhibit neuronal apoptosis, though its *in vivo* effect remains unclear. To address this, this study employed techniques including PCR, tissue fluorescence staining and WB to verify its *in vivo* anti-apoptotic activity: spinal cord neurons were labeled with neuronal markers, apoptotic factors with Cleaved-caspase 3, and changes in the expression of apoptosis-related proteins (Bcl-2, Bax, Cleaved-caspase 3) were detected using immunofluorescence and western blotting. Experimental results showed that both the LA group and HMPB group exhibited certain regulatory and repair effects in downregulating Cleaved-caspase 3 expression and protecting neuronal numbers, which may be related to the basic effects of LA in directly scavenging ROS and HMPB in inhibiting oxidative stress through enzyme-like activity. However, the LA-HMPB combined treatment group showed more prominent advantages, with the lowest fluorescence intensity of Cleaved-caspase 3, the least protein expression and the highest number of surviving neurons (Figure 6A and B). At the mechanistic level, PCR results indicated that compared with the Con group, LA group and HMPB group, the mRNA levels of pro-apoptotic molecules Cleaved-caspase 3 and Bax in the LA-HMPB group were significantly decreased, while the mRNA level of the anti-apoptotic molecule Bcl-2 was significantly increased, suggesting that LA-HMPB may inhibit the initiation of apoptosis in spinal cord tissue by synergistically regulating the mitochondrial apoptotic pathway (Bcl-2/Bax balance) (Figure 6C–E) [37]. This result is consistent with the conclusion from existing studies that ‘antioxidants can inhibit neuronal apoptosis by regulating Bcl-2 family proteins’, and the regulatory amplitude of the combined preparation in this study is more significant, presumably related to the enhanced local concentration of LA by the targeted delivery of HMPB [38]. WB experiments further confirmed that the regulatory effect of the LA-HMPB group on apoptosis-related proteins was superior to that of the LA alone or HMPB alone treatment groups (Figure 6F and G), suggesting that the synergistic effect of the two may not only result from the improvement of physical delivery efficiency but also from the superimposed effects of oxidative stress inhibition and apoptotic pathway regulation. In summary, LA-HMPB provides a new potential strategy for the treatment of SCI through its dual roles in alleviating oxidative stress and inhibiting neuronal apoptosis.

### *In vivo* distribution and excretion of LA and LA-HMPB

**Figure 7:**
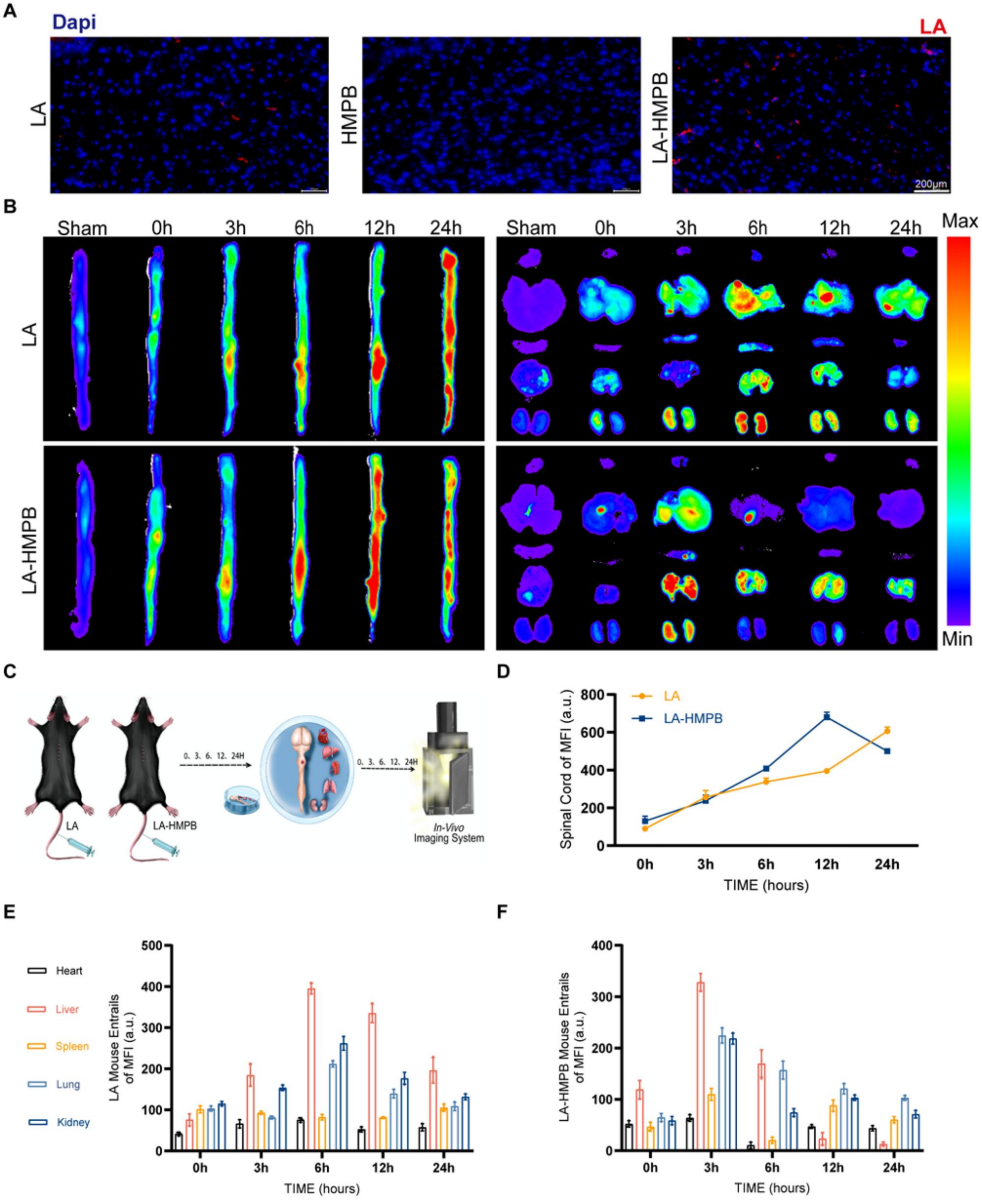
Distribution and excretion analysis of LA and LA-HMPB *in vivo*. (**A**) Differences in LA enrichment levels in the SCI region among LA, HMPB and LA-HMPB groups, scale bar = 200 μm. (**B**) At 3, 6, 12 and 24 h, the drug was administered via the tail vein. The spinal cord, heart, liver, spleen, lung and kidney were imaged. (**C**) The number of LA and LA-HMPB enriched in the SCI region. (**D–F**) Quantitative analysis of fluorescence intensity in the spinal cord and viscera between the LA group and LA-HMPB group. All data are presented as mean ± SD (*n* = 3).

The above experiments showed that it had obvious anti-apoptosis and anti-ROS effects. To investigate the mechanism of action and metabolic efficiency of drugs *in vivo*, we performed tissue fluorescence and *in vivo* imaging experiments. LA was labeled with red fluorescence, images were obtained under a microscope and statistical analysis was performed (Figure 7A; Supplementary Figure S1). The results were consistent with the expectation that LA carrying HMPB was more enriched in the area of SCI [39]. Under the *in vivo* imaging machine, HMPB can deliver LA to the SCI site faster and more accurately [40, 41]. At 6 h, when LA alone had not yet aggregated, LA-HMPB was already enriched to the hit point position. By visceral imaging LA-HMPB is metabolized mainly by the lungs and kidneys and is very fast. In conclusion, it is reasonable to hypothesize that HMPB carrying LA can more easily traverse the damaged blood-spinal cord barrier to accumulate at the injury site, and that its drug metabolism rate is faster, which can effectively reduce its accumulation in the body (Figure 7B–F).

### Lipoic acid-loaded hollow mesoporous Prussian blue nanozyme improves motor function in mice under SCI

**Figure 8:**
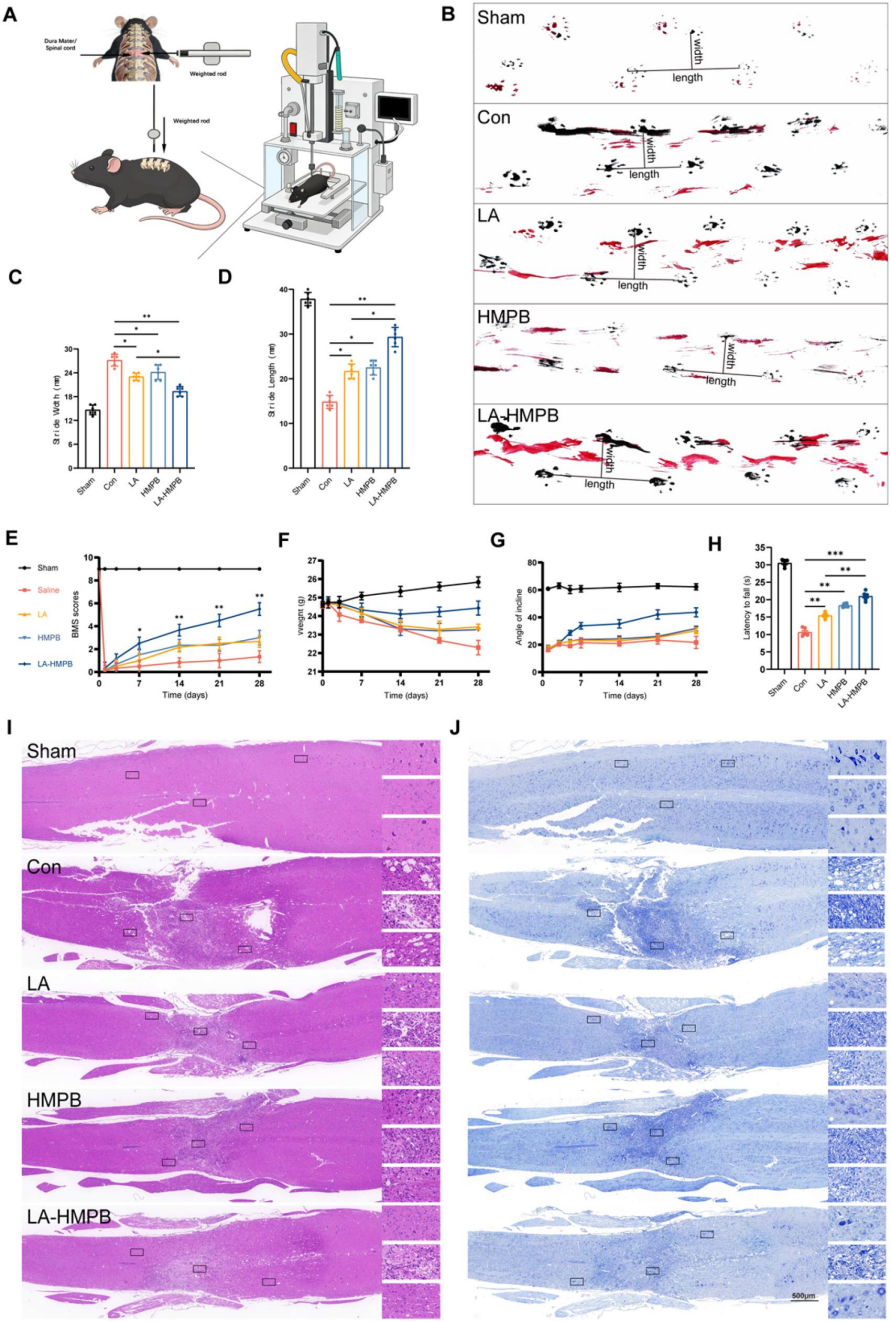
The motor function recovery of mice with SCI in each treatment group was evaluated. (**A**) Schematic diagram of the SCI mouse model establishment process. (**B–D**) Footprints of mice from each experimental group after 28 days, followed by an analysis of stride length and stride width. (**E**) BMS scores of mice from each group recorded on Days 1, 3, 7, 14, 21 and 28. (**F**) Initial body weights of mice and their weights recorded in the first, second, third and fourth weeks for each experimental group. (**G**) Maximum angle observed for mice in each group on Days 1, 3, 7, 14, 21 and 28, representing the inclination angle at which mice could maintain their position on the inclined plate for 5 s. (**H**) The rotarod test assessed motor coordination and balance between groups of mice. (**I** and **J**) Longitudinal sections of spinal cord tissues of mice in each group stained with Nissl and HE and enlarged images of their cephalad ends, injury areas and caudal ends, scale bar = 500 μm. All data are presented as mean ± SD (*n* = 6). *P < 0.05, **P < 0.01, ***P < 0.001.

To assess the therapeutic impact of LA-HMPB on SCI, we administered controlled impacts to the mouse spinal cord as per standard procedures (Figure 8A) [42, 43]. Since motor function recovery post-SCI is a key indicator of therapeutic efficacy, evaluations were conducted using footprint analysis and the BMS score. Motor function in all mouse groups began to recover from the 7th day post-SCI, with the LA-HMPB treatment group showing significantly faster recovery. The LA-HMPB group consistently exhibited superior body balance and motor coordination across all time points compared to other treatment groups (Figure 8B–H). At the time of sampling, the Con group exhibited notable superficial hemorrhage and hematoma compared to the Sham group. Four weeks post-SCI, there was extensive loss of dorsal white matter and central grey matter in the spinal cord’s longitudinal cross-sections. The LA-HMPB group exhibited a greater reduction in lesion area and better preservation of tissue architecture. Additionally, during spinal cord tissue collection, we observed significant inflammatory fibrous tissue surrounding the injured area, leading to spinal cord compression. In the LA-HMPB treatment group, there was a noticeable reduction in inflammatory infiltration, and the spinal stenosis and depression due to compression were partially alleviated (Figure 8I–J; Supplementary Figure S2).

### LA-HMPB activates Keap1/Nrf2 molecular pathway

**Figure 9:**
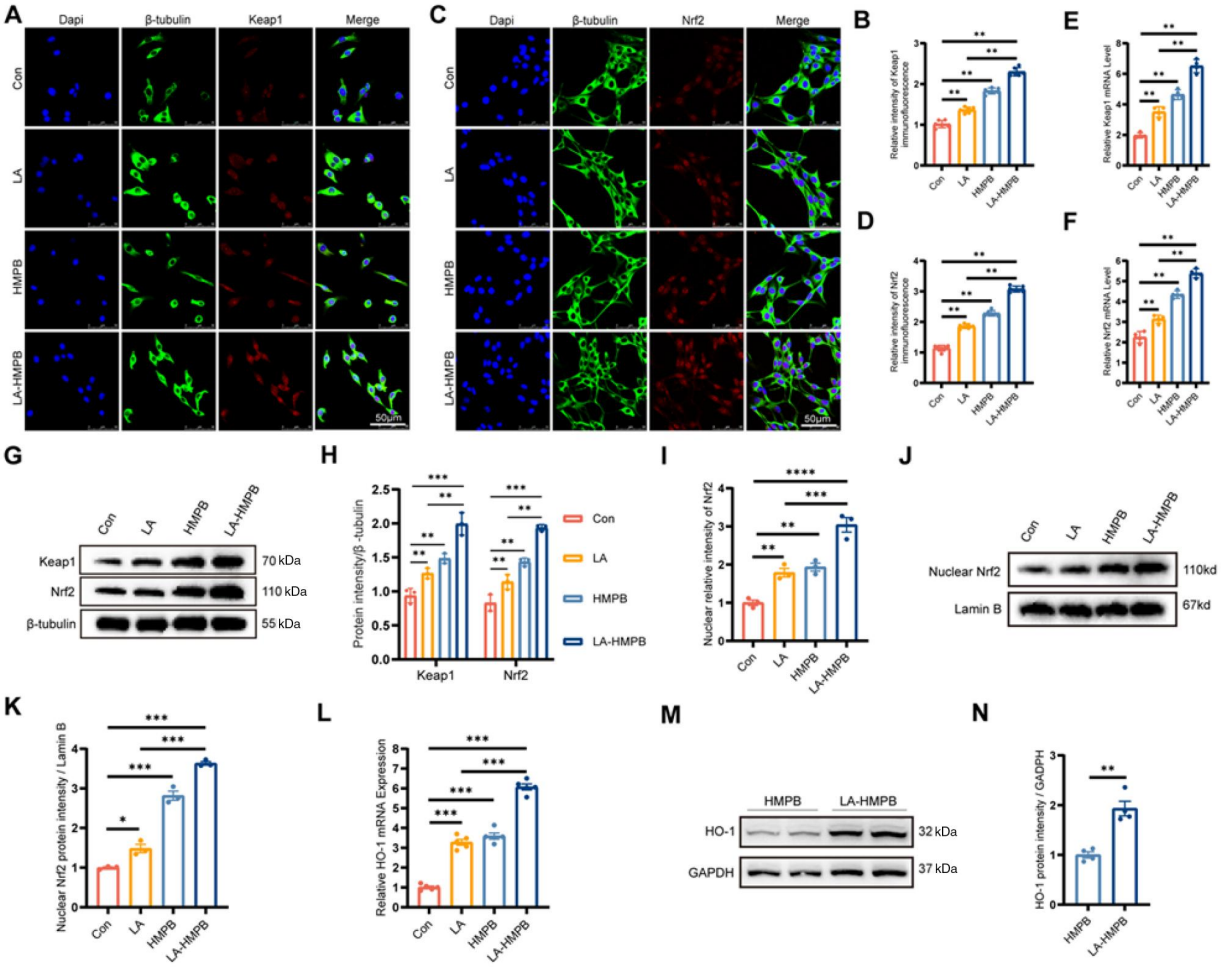
Assessment of Keap1/Nrf2 pathway activation by LA-HMPB. (**A** and **B**) Immunofluorescence analysis of Keap1 expression in PC12 cells of each group, with quantification of fluorescence intensity, scale bar = 50 μm. (**C** and **D**) Immunofluorescence analysis of Nrf2 expression in PC12cells of each group, with quantification of fluorescence intensity, scale bar = 50 μm. (**E** and **F**) Relative Keap1 and Nrf2 mRNA levels in the PC12 cells of each group. (**G** and **H**) Western blot analysis of Keap1 and Nrf2 protein expressions in the PC12 cells of each group, along with quantitative analysis of their grayscale values. (**I**) Immunofluorescence analysis of Nrf2 expression in PC12 cell nucleus of each group, with quantification of fluorescence intensity in (a), ruler = 50 μm. (**J** and **K**) Western blot analysis of Nrf2 protein expressions in PC12 cell nucleus of each group, along with quantitative analysis of their grayscale values. (**L**) Relative HO-1 mRNA levels in PC12 cells of each group. (**M** and **N**) Western blot analysis of HO-1 protein expressions in PC12 cell nucleus of each group, along with quantitative analysis of their grayscale values. All data are presented as mean values ± SD (*n* ≥ 3): *P < 0.05, **P < 0.01, ***P < 0.001.

A series of experimental studies have shown that LA-HMPB has abundant anti-neuronal apoptosis effects. In addition, LA-HMPB also highlights its excellent antioxidant stress effects. Then, the mechanism of LA-HMPB’s antioxidant stress is explored. Studies have identified the Keap1/Nrf2 pathway as essential in the antioxidant stress response [23, 44]. Numerous studies have demonstrated that Keap1 is a crucial antioxidant protein that effectively regulates cellular oxidative stress levels [45, 46]. The Keap1 protein interacts with the transcription factor Nrf2 to regulate the cellular defense mechanism against oxidative stress [47]. Therefore, we validated the results through cellular immunofluorescence experiments. The results were surprising. After incubation in the treatment group, the fluorescent expression of Nrf2 and Keap1 proteins increased to varying degrees, with the LA-HMPB treatment group showing the most significant increase (Figure 9A–D). Subsequently, we homogenized mouse spinal cord tissue to extract RNA and proteins for detection to enhance the accuracy of the experimental results. RT-PCR results showed that the RNA expression levels of Keap1 and Nrf2 in the LA-HMPB treatment group were significantly higher than those in the saline group and were higher than those in the other treatment groups (Figure 9E and F). This conclusion was further confirmed in protein immunoblotting experiments. In comparison to the SCI group, the treatment group showed varying degrees of Keap1 and Nrf2 factors, with LA-HMPB demonstrating the most significant effect (Figure 9G and H). Furthermore, given that Nrf2 translocates into the nucleus upon activation to exert its antioxidant role, we assessed its localization and nuclear expression using western blot and immunofluorescence. Both results demonstrated that LA-HMPB treatment indeed significantly promoted Nrf2 activation and nuclear translocation (Figure 9I–K). To further determine whether the anti-apoptotic effect of LA-HMPB is mediated through the Keap1/Nrf2 pathway, we examined the expression of HO-1, a key downstream antioxidant effector of this pathway. As a result, LA-HMPB was found to markedly upregulate HO-1 expression at both the mRNA and protein levels (Figure 9L–N). Together, these data indicate that LA-HMPB attenuates apoptosis by suppressing oxidative stress via activation of the Keap1/Nrf2 pathway.

## Discussion

SCI can trigger a series of cascade reactions, leading to spinal cord tissue damage and the production of a large number of ROS. When the excessive release of ROS breaks the oxidation-antioxidant balance of the body, it will trigger oxidative stress [48]. Oxidative stress not only directly damages neurons by inducing lipid peroxidation of nerve cell membrane, but also activates intracellular apoptotic signaling pathways, including endogenous pathways mediated by mitochondrial dysfunction and exogenous pathways activated by death receptors, thereby promoting neurons to apoptosis [49].

LA has been widely used in medical and scientific research because of its natural antioxidant properties, especially in the treatment of diabetic peripheral neuritis and tumors, and its safety has been fully verified [50]. However, due to the rapid metabolism of LA in the body, frequent administration is required to maintain the effective concentration of LA, and LA alone is susceptible to the first-pass effect in the liver and has low bioavailability. To achieve an efficient therapeutic effect, we look for nanocarriers for encapsulation to enhance the utilization rate. Nanozymes are widely used in medical treatment, and have good experimental results in anti-tumor therapy, antibacterial application, biosensing and anti-oxidation [51]. For example: regulating ROS scavenging and macrophage repolarization through the HIF-1α/NF-κB cascade for the treatment of rheumatoid arthritis [52]. Multienzymatic activities and antitumor and antibacterial effects of MIL-101(Fe)-derived nanoenzymes cascade [53]. Hollow Cu2MoS4 nanoparticles carrying immune checkpoint inhibitors modify the tumor microenvironment to irregulating ROS scavenging and macrophage repolarization through the HIF-1α/NF-κB cascade for the treatment of rheumatoid arthritis improve immunotherapy outcomes in pancreatic cancer [54]. Probiotics nano-enzyme gel regulates vaginal microenvironment to treat candida vaginitis [55]. The study on engineering immunomodulatory stents with a zinc ion-lysozyme nanoparticle platform for vascular remodeling demonstrated that the HMPB exhibited excellent dispersion, drug loading capacity and diverse nanoenzyme activities [56]. This makes it stand out among the many natural nanozymes. The unique porous structure and high specific surface area of Hollow mesoporous Prussian blue (HMPB) can improve many defects of LA.

The LA-HMPB nano-delivery system designed in this study can efficiently penetrate the cerebrospinal barrier and deliver LA to the lesion of SCI by targeted delivery mechanism, which can significantly improve the efficacy of drug treatment. It is noteworthy that the HMPB carrier itself has a certain therapeutic activity, while the conjugated system of LA and HMPB (LA-HMPB) shows a better synergistic effect in inhibiting oxidative stress injury and neuronal apoptosis than the single component treatment group.

Keap1/Nrf2 signaling pathway plays a key role in maintaining the dynamic balance between peroxides and antioxidants. When the body is in a state of oxidative stress, this pathway is activated and regulates the expression of downstream antioxidant proteins, thereby reducing oxidative damage. Previous studies have confirmed that LA can play an antioxidant role by regulating this signaling pathway. In this study, it was found that LA-HMPB not only retained the biological activity of LA, but also was more significant in the regulatory effect of pathway-related proteins.

In the current development of spinal cord repair strategies, it is urgent to deeply understand the neurobiological cascade and neuronal plasticity mechanism after SCI. Although much remains unknown about the molecular mechanism of neuronal repair, this study demonstrated that HMPB-based nanodelivery system could trigger potent neuroprotective and repair biological activities through the synergistic regulation of oxidative stress-apoptosis signaling network. The therapeutic potential of LA-HMPB conjugate system not only provides a new idea for the clinical transformation of SCI but also is expected to be extended to the repair treatment of other central nervous system injury diseases.

## Conclusions

HMPB prepared from iron sources complexed with LA reveals a green, economical and efficient method for the treatment of SCI. In terms of efficacy, it surpasses traditional HMPB synthesis methods and other widely studied nanomedicines. In the treatment of SCI, compared to HMPB and LA alone, LA-HMPB demonstrates superior therapeutic effects, with higher production efficiency, antioxidant stress response, anti-neuronal apoptosis and superior biosafety. Furthermore, LA-HMPB activates the Keap1/Nrf2 molecular pathway, effectively combating oxidative stress following SCI, thereby inhibiting neuronal apoptosis and promoting the recovery of motor function. Our findings offer therapeutic targets for motor function recovery post-SCI, contributing valuable insights for developing cost-effective and innovative treatments. This also offers a new strategy for enhancing the drug utilization of LA using HMPB, which holds promise for treating oxidative stress responses caused by many diseases.

## Supplementary Material

rbag039_Supplementary_Data
