# Supplementary material for: Functional recovery after spinal cord injury through neuroprotection by lipoic acid-loaded hollow mesoporous Prussian blue nanozymes
Source: Regen Biomater. 2026 Mar 9;13:rbag039. doi: 10.1093/rb/rbag039 (PMC13070658; doi:10.1093/rb/rbag039)
Supplement: rbag039_Supplementary_Data [file rbag039_supplementary_data.zip › supplementary Figure and Table.docx]

**Supporting Information**


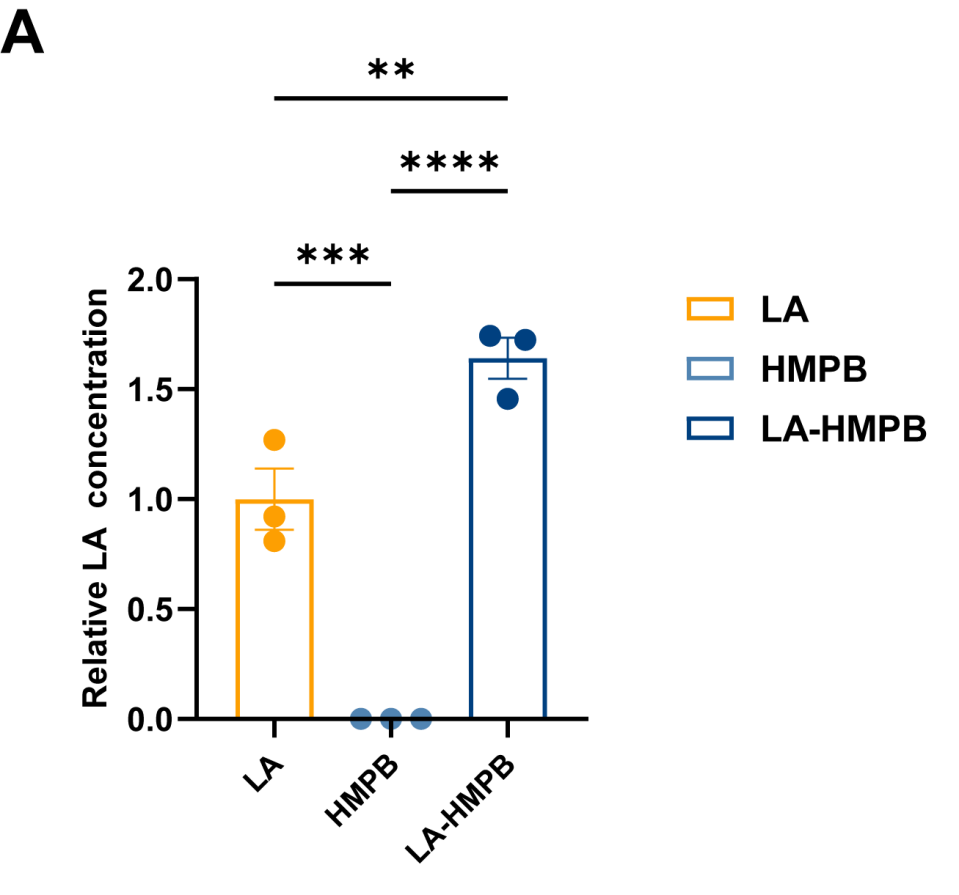


**Figure S1. Comparison of Enrichment Degree of Fluorescently Labeled LA at the Spinal Cord Injury Site Among LA Group, HMPB Group, and LA-HMPB Group.** (A) All data are presented as mean values ± SD (n =3): **p < 0. 05*, ***p < 0. 01*, ****p < 0. 001*.

**
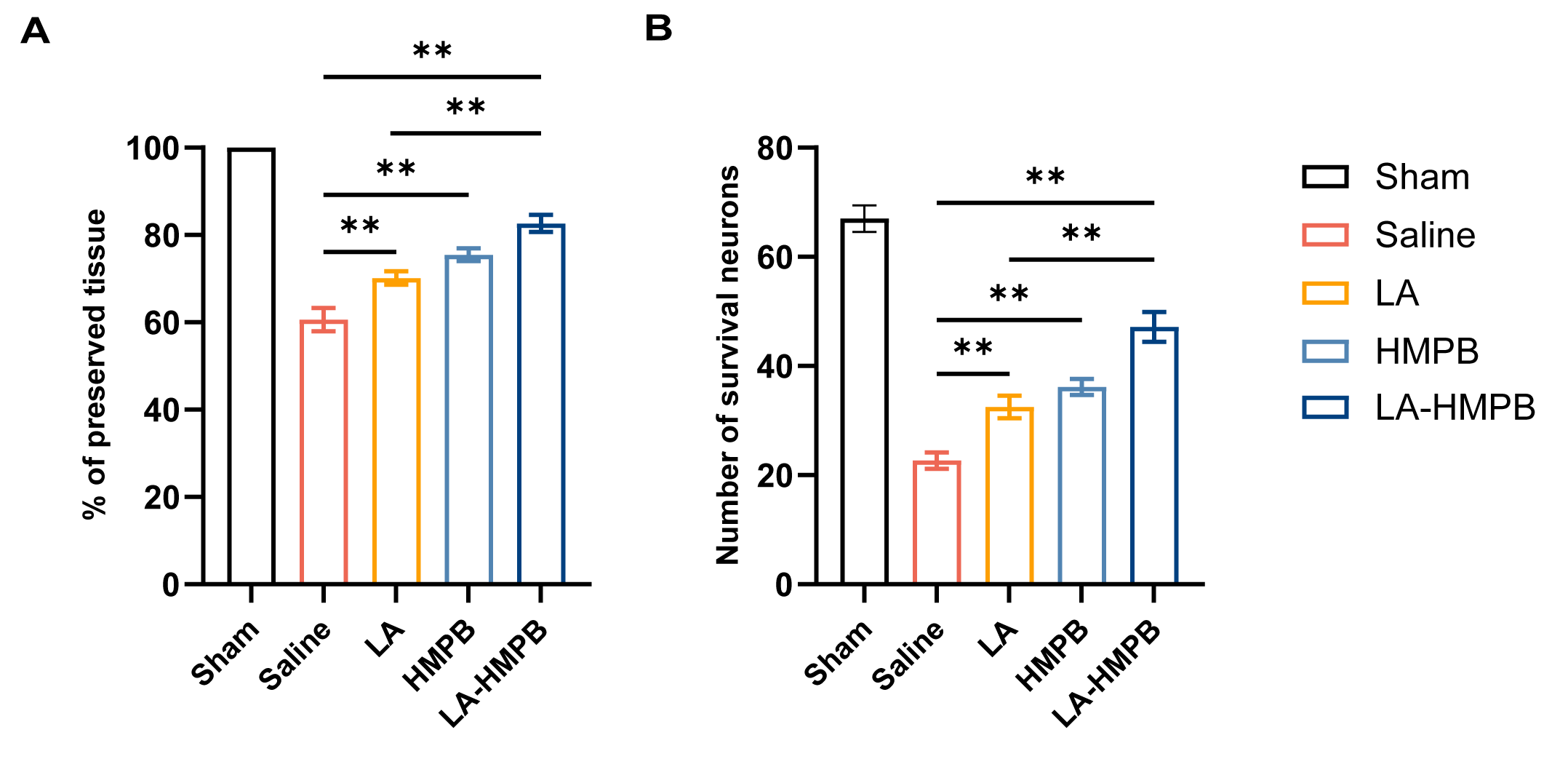
**

**Figure S2. Statistical Quantification of HE and Nissl Staining in Spinal Cord Sagittal Sections.** (A) Statistical Analysis of Lesion Area in HE-Stained sections. (B) Statistical Graph of Neuronal Survival Count in Nissl-Stained.. All data are presented as mean values ± SD (n = 6): **p < 0. 05*, ***p < 0. 01*, ****p < 0. 001*.

**Supplementary Table**

**Table S1. Sequences of Primers for Real-Time Quantitative Polymerase Chain Reaction**

| **gene** | **forward primer sequence (5' -3')** | **reverse primer sequence (5' -3')** |
| --- | --- | --- |
| *Cleaved -caspase 3* | AGCTTGGAACGGTACGCTAA | GAGTCCACTGACTTGCTCCC |
| *BAX* | ATCCAAGACCAGGGTGGCT | CCTTCCCCCATTCATCCCAG |
| *BCL-2* | GAACTGGGGGAGGATTGTGG | GCATGCTGGGGCCATATAGT |
| *KEAP1* | GTGGCACCTACAGAGACACC | GCGCTTGGAGAAGGGCA |
| *NRF2* | AACAGAACGGCCCTAAAGCA | TGGGATTCACGCATAGGAGC |
| *GAPDH* | GGTGAAGGTCGGTGTGAACG | CTCGCTCCTGGAAGATGGTG |
| *HO-1* | CAACATTGAGCTGTTTGAGG | TGGTCTTTGTGTTCCTCTGTC |
